# Potential biomarkers: differentially expressed proteins of the extrinsic coagulation pathway in plasma samples from patients with depression

**DOI:** 10.1080/21655979.2021.1971037

**Published:** 2021-09-07

**Authors:** Chunyue Yu, Teli Zhang, Shanshan Shi, Taiming Wei, Qi Wang

**Affiliations:** a College of Pharmacy, Harbin Medical University-Daqing, Daqing, China; b Department of Pharmacy, The People’s Hospital of Daqing, Daqing, China

**Keywords:** Depression, biomarker, plasma, human, bioinformatics, extrinsic coagulation pathway

## Abstract

Depression is a severe disabling psychiatric illness and the pathophysiological mechanisms remain unknown. In previous work, we found the changes in extrinsic coagulation (EC) pathway proteins in depressed patients compared with healthy subjects were significant. In this study, we screened differentially expressed proteins (DEPs) in the EC pathway, and explored the molecular mechanism by constructing a protein-protein interaction (PPI) network. The DEPs of the EC pathwaywere initially screened by isobaric tags for relative and absolute quantification (iTRAQ) in plasma samples obtained from 20 depression patients and 20 healthy controls, and were then identified by Enzyme-linked immunosorbent assays (ELISAs). Ingenuity Pathway Analysis (IPA) software was used to analyse pathway. The differentially expressed genes (DEGs) were identified by analyzing the GSE98793 microarray data from the Gene Expression Omnibus database using the Significance Analysis for Microarrays (SAM, version 4.1) statistical method. Cytoscape version 3.4.0 software was used to construct and visualize PPI networks. The results show that Fibrinogen alpha chain (FGA), Fibrinogen beta chain (FGB), Fibrinogen gamma chain (FGG) and Coagulation factor VII (FVII) were screened in the EC pathway from depression patient samples. FGA, FGB, and FGG were significantly up-regulated, and FVII was down-regulated. Thirteen DEGs related to depression and EC pathways were identified from the microarray database. Among them NF-κB Inhibitor Beta (NFKBIB) and Heat shock protein family B (small) member 1 (HSPB1) were highly correlated with EC pathway. We conclude that EC pathway is associated with depression, which provided clues for the biomarker development and the pathogenesis of depression.

## 1. Introduction

Depression results from the interactions between social, psychological and biological factors with high prevalence, and high mortality. As an etiologically heterogeneous condition [1], depression has led to an unsatisfactory diagnosis and treatment outcome due to the uncertainty of its pathogenesis and the lack of a clinical diagnosis index [2]. As a result, the chronic and relapsing disease will aggravate the mental and economic burdens of patients and cause complications or even lead to suicide [3]. Therefore, a deeper understanding of the pathogenesis of depression is needed for earlier diagnosis and rehabilitation evaluation of depression.

To date, much progress has been made in the study of the potential etiology of depression, such as the involvement of monoamine neurotransmitters (serotonin, norepinephrine and dopamine) [4], brain-derived neurotrophic factor (BDNF) [5], hypothalamic-pituitary-adrenal (HPA) axis [6], corticotrophin releasing hormone (CRH) [7], chronic systemic inflammation [8], glutamate metabolism [9], neuronal synaptic plasticity [10] and oxidative stress [11] et al. Research showed that serotonin deficiency may lead to the incidence of depression [12], which is currently one of the generally accepted hypotheses. The decrease of norepinephrine and dopamine in the blood and cerebrospinal fluid of patients with depression suggests a relationship between depression and monoamine neurotransmitters [13,14]. Plasma brain-derived neurotrophic factor levels were significantly reduced in depression [15]. Kudinova [16] et al. demonstrated that brain-derived neurotrophic factor inhibits the cascade of cell death by activating the MAPK pathway to protect neurons, producing an antidepressant effect. In addition, it is generally accepted that the cortisol levels and corticotrophin releasing hormone concentration in the plasma of depression patients were higher than those in the control group, which suggested that the hyperactivity of the hypothalamic-pituitary-adrenal axis is associated with depression [17–19]. Increased inflammatory markers have been found in depression, and the inhibition of inflammatory factors can reduce the depressed mood [20–22]. Many studies have found variations in the levels of glutamate and its metabolism in different brain regions in patients with depression by magnetic resonance spectroscopy (MRS) [23–25]. Lilly Schwieler [26] et al. used electroconvulsive therapy (ECT)on treatment-resistant depressed patients and found that an antidepressant effect can be achieved by inhibiting the neurotoxicity branch of the kynurenine pathway. As a hypothesis, each viewpoint lacks clinical applicability, sensitivity and specificity [27]. Even labeled antidepressants (such as 5-hydroxytryptamine reuptake inhibitors) have problems, such as slow effect, and can be ineffective for some patients. Therefore, it is necessary to explore the pathogenesis of depression.

In previous work [28], we adopted the iTRAQ based quantitative proteomic method to identify 153 DEPs in plasma samples from healthy control subjects (n = 22) and depression patients (n = 20). Using IPA software for the analysis of the disease, as well as the functions and pathways of the differential proteins, it was found that cardiovascular disease had the highest correlation with depression. There is a close relationship between cardiovascular disease and the EC pathway [29]; therefore, we chose this pathway to study. Studies have confirmed that depression is one of the risk factors of cardiovascular disease, which may affect the prognosis of cardiovascular diseases through platelet dysfunction, autonomic dysfunction and an abnormal immune response [30]. The adverse effects of cardiovascular disease can also lead to depression [31], as they promote each other. Whether the abnormality of the EC pathway is related to the pathogenesis of depression and whether it is the key factor of the conversion between depression and cardiovascular disease, have yet to be determined. These limitations inspired this study. Studies have shown that the Hamilton Depression Scale (HAMD) scores of depression patients were positively correlated with fibrinogen content [32], and elevated platelet activity was associated with depression in children [33]. Therefore, the intrinsic relationship between depression and the EC pathway merits further study.

The Human Protein Reference Database (HPRD) (http://www.hprd.org/) contains manually curated scientific information pertaining to the biology of most human proteins, is the largest human PPI database for document mining. Studying PPI networks through bioinformatics can reveal protein interactions that are important for the discovery of the target proteins and the pathogenesis of the disease [34]. We obtained the DEGs by comparing gene chips of depression patients with healthy people, then mapped the DEPs we found in the EC pathway and the DEGs to HPRD PPI networks to establish a sub-network of them, to study the relationship between the nodes to further explore the possible molecular mechanisms of EC pathway involved in the regulation of depression, and to provide a new theoretical support for the clinical study of depression. In addition, scientists have used cerebrospinal fluid [35], brain tissue (autopsy) [36] and peripheral blood samples [37] to study depression. Due to the invasiveness of biopsy and puncture and the limitations of autopsy, the first two materials are not the most suitable for depression. While peripheral blood has a universal applicability for its convenience as well as minimal injury [38]. The contents of the peripheral blood and cerebrospinal fluid can be exchanged through the blood-brain barrier, and since there is 500 ml of cerebrospinal fluid exchanged in blood every day [39], we chose peripheral blood as the research material.

## 2. Materials and methods

### 2.1. Subjects and ethics statement

A total of 20 first-episode depression patients (including 8 treatment patients) were recruited from the psychiatric center of the Third Hospital in Daqing City, Heilongjiang Province, China. 20 healthy controls were recruited at Harbin Medical University-Daqing Campus. Patients were diagnosed according to the Fourth Edition of the Diagnostic and Statistical Manual of Mental Disorders by a psychiatrist using the Mini International Neuropsychiatric Interview [40]. The depression severity level was measured using the 17-item Hamilton Depression Rating Scale [41], and patients with scores over 17 were included. Exclusion factors included the following [42] patients with other comorbid psychiatric disorders (such as schizophrenia, psychotic disorder, or chronic fatigue syndrome); patients who had taken psychotropic medications (antidepressants, anxiolytics, etc.) during the past 8 weeks; healthy control subjects who previously had depression or had a family history of psychiatric disorders; any participants taking a drug (such as non-steroidal anti-inflammatory drugs) that affects the drug concentration in the blood; patients suffering from cardiovascular disease, hypertension, metabolic diseases or who were pregnant, menstruating or on a care period.

We were approved prior to the study by the Medical Ethics Committee of Harbin Medical University (China), and an informed consent agreement was obtained from all participants before study initiation. This study adheres to the latest version of the Declaration of Helsinki.

### 2.2. Plasma sample collection

Blood samples (5 ml) were drawn from the antecubital vein between 7:00 and 9:00 a.m. and incubated at room temperature for 20 min for blood coagulation, followed by centrifugation at 2000 × g for 20 min at 4°C. Then, the supernatants were stored at −80°C until analysis [43].

### 2.3. Extrinsic coagulation pathway analysis

In previous works [28], 153 DEPs have been found among 20 depression patients and 20 healthy controls by the iTRAQ method, according to the IPA software (IPA software v7.1, Ingenuity System Inc., Redwood City, CA, USA; www.ingenuity.com). For the disease analysis results, the EC pathway was selected as the research target. In this experiment, we used IPA software to draw the pathway chart, and the DEPs were briefly analyzed.

### 2.4. Verification of differentially expressed proteins by ELISA

Commercially available sandwich ELISA kits (Shanghai Bioleaf Biotechnology Co. Ltd., Shanghai, China) were used to detect the content of DEPs such as FGA (abx515963), FGB (abx516323), FGG (abx253654), FVII (EKC33175), FX(abx514145), FXII(abx151465), FII (abx151106), SERPINC1 (abx514280), PROC(abx350615) and PROS1(abx518986) in 40 plasma samples. The protocol was followed according to the manufacture’s instructions. A microplate reader (DNM-9602, Perlong Medical, Jiangsu, China) was used to determine the absorbance of the standards and samples at 450 nm. The intra- and inter-day variations were < 8%, and the concentrations of the samples were calculated according to a standard curve.

### 2.5. Bioinformatics analysis. Data source

The gene expression profile of GSE98793 was obtained from the National Center for Biotechnology Information (NCBI) GEO database (http://www.ncbi.nlm.nih.gov/ geo). A total of 128 specimens, including 64 control group and 64 depressions without anxiety disorder, were evaluated in this data set [44].

### 2.6. Data preprocessing

The original data were pre-processed with R language by BiocGenerics, Biobase, Splines, multtest, siggenes packages and the original CEL files were converted to probe expression profiles. Then we use the SAM statistical method to identify the DEGs between depression and healthy people (Fold change > 1.2 or < 0.83, and p < 0.05).

### 2.7. PPI network construction and subnetwork mining [45]

**Figure 1.**
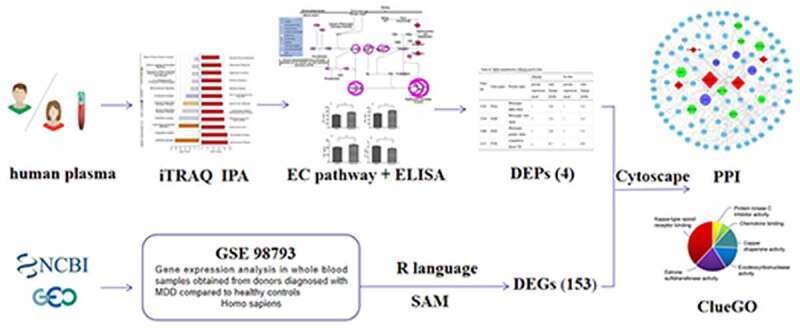
Experimental process for the study of the EC pathway in patients with depression

The PPI network in the HPRD database was downloaded and visualized using Cytoscape version 3.6.0. The DEGs and DEPs were mapped into the PPI network, first neighbors of both them were extracted to form a sub-network. The nodes with degree≤5 in the subnetwork were discarded, thus forming a PPI network map related to the DEPs in the EC pathway of depression patients. Then the molecular functions of the DEGs were analyzed using the ClueGO plug-in. The experimental design is shown in Figure 1.

### 2.8 Statistical analysis

The ELISA data are expressed as the mean ± SD, and p < 0.05 was considered statistically significant. Between-group comparisons were analyzed by the Student’s t-test. All statistical analyses were performed using Graph Pad Prism (Version 5.0, Graph Pad Software, CA, USA).

## 3. Results

By studying the differentially expressed proteins of the exogenous coagulation pathway in the plasma samples of depression patients, we found that FGA, FGB, FGG and FVII in the EC pathway have all changed significantly. The PPI results showed that NFKBIB and HSPB1 had the highest correlation with the differentially expressed proteins in the EC pathway.

### 3.1. Subject characteristics

The detailed demographic and clinical characteristics of the recruited subjects are summarized in Table 1. The mean (SD) ages of the depression patients and healthy controls were 45.05 (2.17) and 43.40 (2.15), respectively. There were no significant differences between the two groups in terms of their demographic characteristics, including age, sex and body mass index (BMI) (p > 0.05).

**Table 1.** Demographic and clinical characteristics of recruited subjects

Variables	Depression patients		
(N = 20)	Healthy controls		
(N = 20)	P-value^a^		
Sex(M/F)	8/12	9/11	1
Age(years) ^b^	45.05 ± 2.17	43.40 ± 2.15	0.97
BMI^b^	22.09 ± 0.36	22.66 ± 0.43	0.43
HDRS scores^b^	20.35 ± 2.39	5.37 ± 1.12	0.00

^a^Two-tailed Student’s t-test for continuous variables (age, BMI, and HDRS scores); Chi-square analysis for categorical variables (sex).

^b^Age, BMI and HDRS scores are presented as the means ± SD. Abbreviations: M, male; F, female; BMI, body mass index; HDRS, Hamilton depression rating scale.

### 3.2. Screening of DEPs of the EC pathway

The EC pathway is the main pathway of blood coagulation. When the body experiences inflammation or tissue damage, tissue factor (TF) and the activated FVII (FVIIa) form TF-FVIIa complex. Then, the complex binds to activated FXa in the presence of phospholipid membranes and calcium ions, and further binds to FVa, forming a TF-FVIIa-FXa-FVa complex through the thrombin cascade, which changes the prothrombin to thrombin. Under the action of thrombin, fibrinogen becomes a fibrin monomer, which further generates blood clots and completes the clotting process. When the body is in the coagulation state for a long time, thrombin will activate the production of anticoagulant substances such as protein C (PROC), protein S (PROS), antithrombin III (ATIII), tissue factor pathway inhibitor (TFPI), etc., and regulate the balance of coagulation and anticoagulant system through negative feedback.

**Figure 2.**
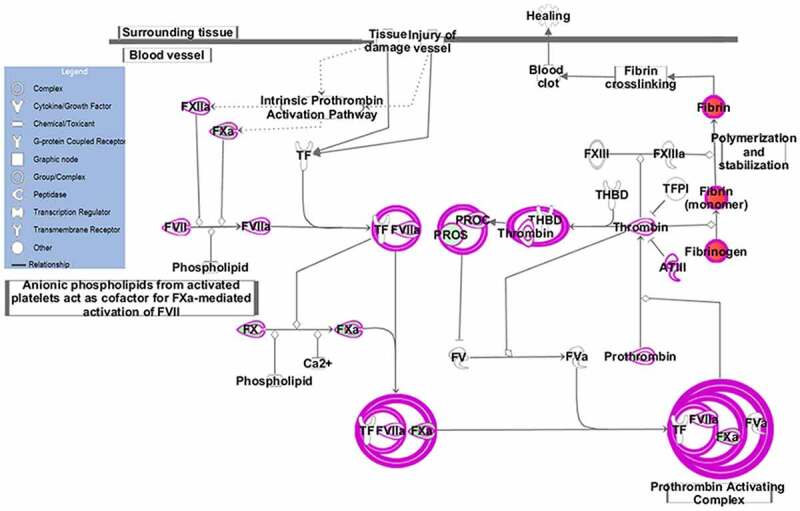
EC pathway. The EC pathway was constructed based on IPA mapping. Green represents the down-regulated proteins, red represents the up-regulated proteins and pink circles indicate the complex formed during coagulation

**Figure 3.**
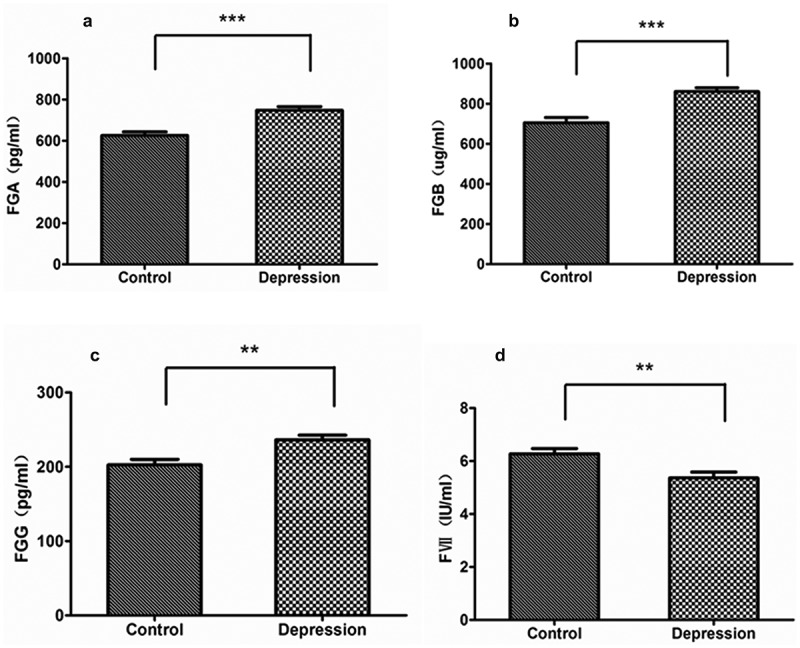
ELISA validation of DEPs in the EC pathway from depression patients (n = 20) and controls (n = 20). (a) FGA. (b) FGB. (c) FGG. (d) FVII. Data are expressed as mean ± s.e.m. p values were obtained by Student’s t-test statistical analysis. *p < 0.05, **p < 0.01, ***p < 0.001. Acronyms: FGA, fibrinogen alpha chain; FGB, fibrinogen beta chain; FGG, fibrinogen gamma chain; FVII, coagulation factor VII

In our previous work, we used the iTRAQ method to find 10 DEPs in the EC pathway: FGA, FGB, FGG, FVII, FX, FXII, FII (PT), SERPINC1 (AT-III), PROC and PROS1. In this study, the pathway diagram was created using IPP software (Figure 2). Down-regulated proteins such as FⅦ, FⅩ, FⅫ, FII (PT), SERPINC1 (AT-III), PROC and PROS1 are shown in green, and up-regulated proteins such as FGA, FGB and FGG are shown in red. Pink circles indicate the complex formed during coagulation. Then we use the ELISA method to verify the DEPs (Table 2). Among the DEPs, 4 proteins were consistent with the results of iTRAQ and had statistical significance (p < 0.05): FGA, FGB, FGG and FVII (Figure 3). To the best of our knowledge, the association between FGA and FVII and depression has been reported [46,47], while FGG and FGB have not been reported. Compared with the healthy group, the levels of FGA (p < 0.001), FGB (p < 0.001) and FGG (p < 0.01) were significantly increased in the depression group, and FVII (p < 0.01) was significantly decreased. Although the FX validation results were consistent with the iTRAQ results, they were not statistically significant and therefore were excluded. The remaining proteins were also excluded because their trend changes were inconsistent with the iTRAQ results. Therefore, we have obtained four differential proteins in the EC pathway that are associated with depression by using the two methods of iTRAQ and ELISA.

**Table 2.** DEPs identified by iTRAQ and ELISA

Gene ID	Gene name	Protein name	iTRAQ	ELISA		
			protein expression trend	fold change		
(D/H)	protein expression trend	fold change				
(D/H)						
2243	FGA	fibrinogen alpha chain	↑	2.6	↑	1.2
2244	FGB	fibrinogen beta chain	↑	2.4	↑	1.2
2266	FGG	fibrinogen gamma chain	↑	2.6	↑	1.1
2155	FⅦ	coagulation factor Ⅶ	↓	0.7	↓	0.9
2159	FⅩ	coagulation factor Ⅹ	↓	0.4	↓	0.9
2161	FⅫ	coagulation factor Ⅻ	↓	0.7	↑	1.2
2147	FII(PT)	coagulation factor II	↓	0.9	↑	1.0
462	SERPINC1					
(AT-III)		serpin family C member 1	↓	0.7	↑	1.3
5624	PROC	protein C	↓	0.6	↑	1.4
5627	PROS1	protein S (alpha)	↓	0.6	↑	1.2

Abbreviations: D, depression patients; H, healthy controls.

### 3.3. Results of bioinformatics analysis

**Figure 4.**
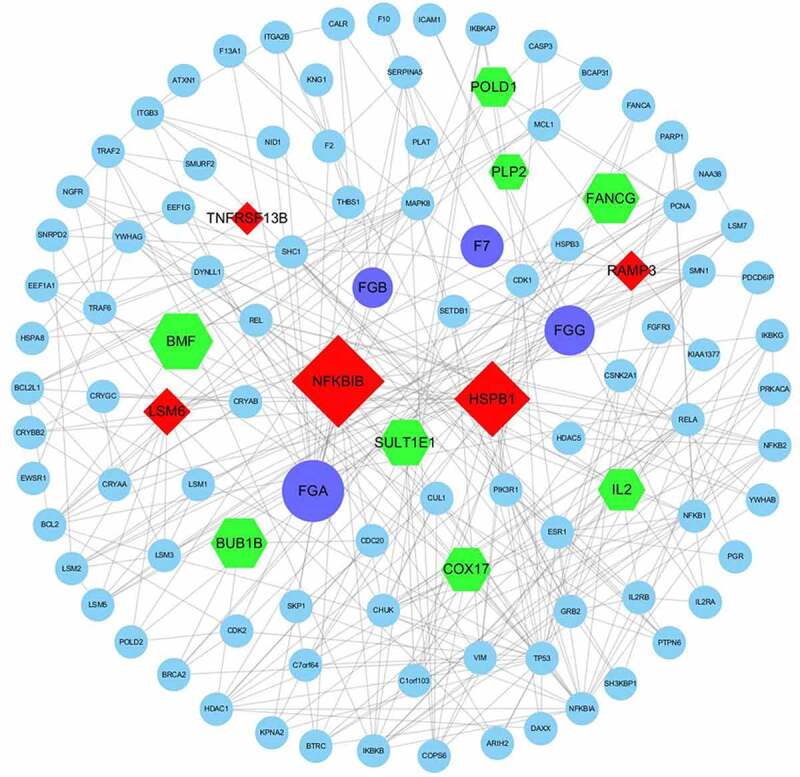
PPI sub-network obtained by Cytoscape. Purple – DEPs in the EC pathway verified by ELISA; red – upregulated DEGs; green – downregulated DEGs; blue – other proteins; lines – relationships

After preprocessing, 153 DEGs (Fold change > 1.2 or < 0.83, and p < 0.05) in the depressive patient chip were screened out. Then the 153 DEGs and 4 DEPs (FGA, FGB, FGG, FVII) were mapped into the PPI network from HPRD database, first neighbors of both them were extracted to form a PPI sub-network. In the sub-network, each node represents a protein or gene, and each line represents an association between them. Nodes that were not associated with other nodes were deleted, and nodes with a degree of association less than or equal to 5 were also deleted. The results of the PPI sub-network diagram were shown in Figure 4. Here, purple nodes represent the DEPs in the EC pathway verified by ELISA, both red and green are differential genes from the gene chip of patients with depression. Red represent up-regulated DEGs, green represents down-regulated DEGs, and blue represent other proteins that interact. It can be seen from the figure that the four DEPs we verified have a direct or indirect relationship with the DEPs in the patient’s chip of depression, and may also act through other proteins, thus demonstrating the correlation between the DEPs in the EC pathway and depression. The size of the DEPs and the DEGs in the figure were determined by the ‘degree’ (the size of other proteins has nothing to do with the ‘degree’, set to the same size). The larger the ‘degree’ of nodes, the more nodes in the PPI network was related to the proteins or genes.

As shown in the figure, there were 13 DEGs interacting with the DEPs found in the EC pathway in the PPI network, with 5 up-regulated (TNFRSF13B, NFKBIB, HSPB1, LSM6, RAMP3) and 8 down-regulated (SULT1E1, COX17, FANCG, BUB1B, IL-2, POLD1, PLP2, BMF). The results were shown in Table 3. As the top genes of the ‘degree’, it was clear that the role of NFKBIB and HSPB1 in the PPI network was more critical and we will discuss it later.

**Table 3.** The DEGs that interact with DEPs in the EC pathway. P-values are the average of two technical replicates calculated from the raw data

Gene Symbol	Full name	Fold Change	p value	q value	Degree
TNFRSF13B	TNF receptor superfamily member 13B	1.353296	3.17E-05	0.089379	6
NFKBIB	NFKB inhibitor beta	1.323393	0.014847	0.324840	29
HSPB1	heat shock protein family B (small) member 1	1.310663	0.000262	0.130834	22
LSM6	LSM6 homolog	1.251765	0.006701	0.261199	10
RAMP3	receptor activity modifying protein 3	1.202286	0.003357	0.217228	7
SULT1E1	sulfotransferase family 1E member 1	0.828337	0.000597	0.155498	12
COX17	cytochrome c oxidase copper chaperone COX17	0.825958	0.016704	0.330279	11
FANCG	FA complementation group G	0.818217	0.004445	0.240950	14
BUB1B	BUB1 mitotic checkpoint serine/threonine kinase B	0.811936	0.001696	0.181073	14
IL-2	interleukin 2	0.793038	0.001729	0.181341	10
POLD1	DNA polymerase delta 1, catalytic subunit	0.792027	0.004811	0.244754	9
PLP2	proteolipid protein 2	0.789695	0.000138	0.099669	7
BMF	Bcl2 modifying factor	0.785858	0.001491	0.171715	6

**Figure 5.**
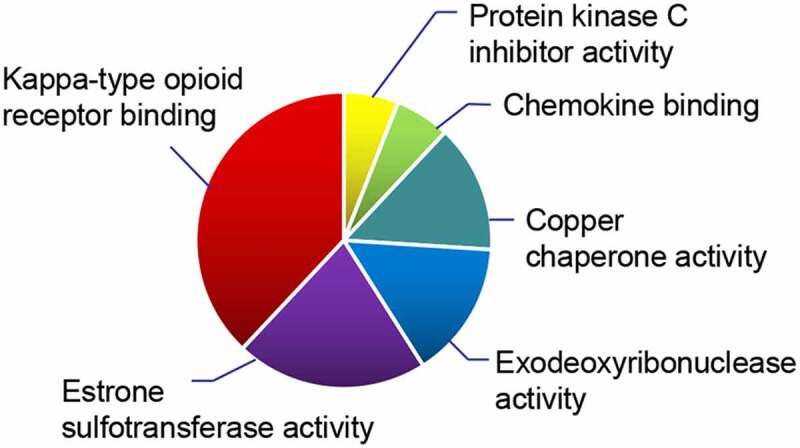
ClueGO results. Molecular function

The molecular functions of the 13 DEGs were analyzed using the ClueGO plug-in to reveal possible mechanisms of action (Figure 5). We found they were mainly involved in the Kappa-type opioid receptor binding, estrone sulfotransferase activity, exodeoxyribonuclease activity and copper chaperone activity.

## 4. Discussion

Based on the previous work, this article screened four key proteins in the EC pathway of depression patients by iTRAQ and ELISA. Among them, the study on the correlation between FVII, FGA and depression has been reported [46,47], but FGG and FGB have not been reported yet. We verified the correlation between the four proteins and depression using bioinformatics methods. A preliminary analysis of its mechanism of action was conducted. The result of this paper will provide strong theoretical support for the development of potential biomarkers for depression and the etiology of depression.

We found that compared with healthy people, the EC pathway was abnormal in patients with depression. In particular, the changes of F FVII, FGA, FGB and FGG in pathways were very obvious. FGA, FGB and FGG were up-regulated and FVII was down-regulated. FGA, FGB, and FGG are the three polypeptide chains that make up fibrinogen. As key proteins in the end of EC pathway, an increase in FGA, FGB and FGG indicate that the pathway is activated and the body has developed a hypercoagulable state. This result is consistent with previous literature reports. Literature showed that increased depressive symptoms were significantly associated with fibrinogen levels [48] and there was a positive correlation between the Hamilton Depression Scale score and the content of fibrinogen in patients with depression [32]. A large survey of 73,367 individuals found that elevated levels of fibrinogen in the plasma was associated with psychological distress, use of antidepressants, and increased hospitalization for depression [49]. D Martins-de-Souza [46] found that the levels of FGA in depression patients were significantly higher than those in the normal group and that FGA decreased significantly after treatment, suggesting that FGA could be a potential biological target for depression. In addition, fibrinogen is closely associated with adverse cardiovascular events, which are both an independent risk factor for coronary heart disease and a risk factor for transient ischemic attack and cerebral infarction [50]. Studies have shown that the presence of depression significantly increases the incidence of cardiovascular disease [51–53], which is a common complication of hypertension [54], coronary artery disease [55], vascular dementia [56] and stroke [57]. The adverse prognosis of cardiovascular disease also contributes greatly to the onset of depression [58]. Abnormal expression genes of the EC pathway have been recognized for their association with cardiovascular disease [59], and in the present study, abnormalities of the EC pathway were also shown in patients with depression. In combination with the close association between depression and cardiovascular disease, we can speculate that EC pathways, as the confluence of two diseases, may be involved in the pathogenesis of depression and may also lead to the transformation of the two diseases, although the specific mechanism merits further study and discussion.

FVII is one of the promoters of the EC pathway. It is an essential vitamin K dependent factor and a serine protease in hemostasis [60]. In previous studies, F7 has increased in depression. Studies have shown that depression in patients over the age of 65 was associated with elevated levels of factor FVII and fibrinogen [47]. Depressive mood is related to the hypercoagulable state caused by the increase of coagulation factors FVII and FX [61]. Yang found that FIII, FV, and FVII in patients with depression accompanied by suicidal behavior were both elevated and FX decreased, suggesting that the suicidal behavior of depression is closely related to the activation of EC pathway [62]. However, in our study, FVII was found to be reduced. The reason may be that the continuous activation of the EC pathway leads to the initiation of anticoagulant system in the body, resulting in the inhibition of serine protease inhibitors or tissue factor pathways, and inactivating FVII eventually leads to its reduction. Or the level of FVII is related to the age of depressive patients or the type of depression. This hypothesis needs further confirmation.

We also conducted further research on the key proteins found using bioinformatics methods. We use the protein data in the HPRD database as the basis because there are more than 30,000 interacting protein data in the database and it is the most comprehensive text mining database related to human genes and proteins. By mapping the DEPs of the EC pathway we screened and the DEGs of depression screened by the microarray into the PPI network, the first-neighbors was extracted and the subnetwork was establishment, forming a PPI network related to both depression and the EC pathway. In the network we can discover the potential relationship between these DEPs and DEGs, thus demonstrating the relevance of the EC pathway and depression, and provide clues for further study of the mechanism of action. In the PPI subnetwork we constructed, we found that there are multiple DEGs that interact with the DEPs. Here we focus on the first two genes that are ranked far ahead of the relationship, namely NFKBIB and HSPB1.

NFKBIB (NF-κB Inhibitor Beta), also known as ikappabeta or iκbβ. The protein encoded by this gene belongs to the NF-κB inhibitor family and inhibits NF-κB by forming complexes and trapping them in the cytoplasm [63]. When the serine residues on these proteins are phosphorylated by kinase, NF-κB is activated and translocates to the nucleus to function as a transcription factor [64]. When NF-κB is activated to a certain extent, it will up-regulate the gene expression of NFKBIB to inhibit the activity of NF-κB, which is a form of negative feedback regulation [65,66]. Studies have shown that NF-κB is involved in important processes such as inflammation, immune response and apoptosis, and is closely related to various central nervous system diseases such as depression [67,68], Alzheimer’s disease [69] and Parkinson’s disease [70,71]. NF-κB is activated in all of the above conditions, whereas persistent activation causes delayed neuronal death.

The PPI results of this study show that abnormalities of EC pathways in depression are highly correlated with the up-regulation of NFKBIB. As mentioned earlier, up-regulation of NFKBIB leads to over-activation of NF-κB, and activation of NF-κB is a central link in the inflammatory response, leading to increased secretion of cytokines and inflammation. The inflammation hypothesis has been widely recognized by the public as one of the hypothesis of the onset of depression [72–74]. The inflammatory response has a network relationship with the coagulation process. Coagulation promotes inflammation [75], and natural anticoagulants have anti-inflammatory effects [76]. In the EC pathway, when TF is combined with FVII, it can cause intracellular Ca^2+^ influx, phosphorylation of intracellular proteins and activation of related signaling pathways. Thus, it can participate in inflammatory response and induce the inflammatory process and the initiation of exogenous coagulation. Therefore, we boldly speculated the results of this experiment: the interaction between up-regulation of NFKBIB and over activation of NF-κB forms a vicious circle, eventually triggering inflammation, and inflammation activates the EC pathway, both of which contribute to involve in the mediation of depression.

HSPB1 (Heat Shock Protein Family B (Small) Member 1), also known as HSP27, is a member of the heat shock protein family and is a small-molecule heat shock protein commonly found in eukaryotic cells and serves as an ATP-independent molecule partner [77]. It has been at low expression levels under normal physiological conditions. However, when the cells are stimulated by various factors such as oxidative stress and high temperature, expression is up-regulated, and they rapidly phosphorylate and enter the cell nucleus to function to protect cells [78]. HSPB1 has neuroprotective and anti-apoptotic functions and is not only frequently reported in cancers [79,80], but also has a close relationship with central nervous system diseases. HSPB1 is significantly up-regulated in the cortex of Alzheimer disease [81,82] and is also significantly increased in the cortex of Parkinson’s patients [83]. Renkawek K et al. [84] found that HSPB1 immunoreactivity was only observed in hippocampal protein extracts in the status epilepticus group and was therefore considered to be a biomarker of epilepsy. Trystuła M [85] demonstrated that post-stroke depression is associated with HSPB1 over expression. This experiment found that the abnormality of EC pathway in depression patients is highly correlated with the up-regulation of HSPB1. We speculate that oxidative stress may be an important factor in causing changes in both. There is a direct relationship between stress and depression, and it has been widely agreed that the use of unpredictable stress to build animal models of depression. Acute stress will cause the levels of coagulation factors VII, VIII, XII and fibrinogen, platelets, von Willebrand factor antigen, and plasminogen activator to be up-regulated [86]; chronic stress may inhibit the fibrinolytic pathway, Long-term blood hypercoagulation of the body, increasing the risk of thrombosis [87]. Although there is less research on the relationship between HSPB1 and depression, Heat shock protein 70 (HSP70) in the same family has been shown to be closely related to depression. Studies have shown that [88] genetic variations in genes encoding HSP70 family proteins may influence the effects of antidepressants and thus their therapeutic effects. Allele-specific aberrant transcripts of the HSP70 gene on chromosome 6 may be the basis for altered stress and/or immune responses in major depression [89]. Therefore, based on the study of this experiment, it will be of great significance to further study the mechanism of action of HSPB1 and depression.

GlueGO results showed that the molecular functions of the 13 DEGs screened in the PPI network map were mainly concentrated on Kappa-type opioid receptor (KOR) binding. A large number of studies have shown that KOR system is involved in the pathophysiology of affective disorders, drug addiction and depression. The use of KOR agonists in animals and humans can produce anxiety and depression-like effects [90] whereas KOR antagonists demonstrate a reliable antidepressant effect in animal models [91]. Although no KOR antagonists are currently in clinical use, formulations such as buprenorphine, ALKS5461, and CERC-501 are in clinical trials and have good antidepressant effects [92]. It is believed that in the future, short-acting KOR antagonists may be developed to treat depression.

Depression has always been a research hotspot in the medical field. In addition to using bioinformatics technology [2], we also use iTRAQ and ELISA double verification methods, the results obtained are more scientific and credible. There are several limitations to the present study. First, there is no distinction between disease samples, including both drug patients and free-drug patients, possibly impacting the final results. Second, the sample size is relatively small, which may raise the risk of false positive results. Third, the specific mechanism of the EA pathway on depression is not clear, and further investigations are critical to understanding the underlying pathophysiology of depression.

## 5. Conclusions

In conclusion, we screened 4 DEPs (FGA, FGB, FGG and FVII) in the EC pathway from depression patients’ plasma and provided potential biomarkers for the diagnosis of depression. Furthermore, we speculate that NFKBIB and HSPB1 play an important role in the relationship between these differential proteins and depression.

## Highlights

1. Extrinsic coagulation pathway is closely related to depression.
2. FGA, FGB, FGG and FVII are expected to be potential biomarkers of depression.
3. NFKBIB and HSPB1 play an important role between EC pathway and depression.
